# 3D-image-guided high-dose-rate intracavitary brachytherapy for salvage treatment of locally persistent nasopharyngeal carcinoma

**DOI:** 10.1186/1748-717X-8-165

**Published:** 2013-07-05

**Authors:** Yu-Feng Ren, Xin-Ping Cao, Jia Xu, Wei-Jun Ye, Yuan-Hong Gao, Bin S Teh, Bi-Xiu Wen

**Affiliations:** 1 State Key Laboratory of Oncology in Southern China, Department of Radiation Oncology, Cancer Center, Sun Yat-sen University, 651 Dongfeng Road East, Guangzhou 510060, China; 2 Department of Radiation Oncology, The First Affiliated Hospital, Sun Yat-sen University, 58 Zhongshan Rd II, Guangzhou 510080, China; 3 Division of Emergency Medicine, First Affiliated Hospital, Sun Yat-sen University, Guangzhou 510080, China; 4 Department of Radiation Oncology, The Methodist Hospital, 6565 Fannin, DB1-077, Houston, Texas 77030, USA; 5 The Methodist Hospital Research Institute, 6565 Fannin, Houston, Texas 77030, USA

**Keywords:** Nasopharyngeal carcinoma, Intensity-modulated radiotherapy, Persistent disease, 3D-image-guided HDR Brachytherapy, Local tumor control

## Abstract

**Background:**

To evaluate the therapeutic benefit of 3D-image-guided high-dose-rate intracavitary brachytherapy (3D-image-guided HDR-BT) used as a salvage treatment of intensity modulated radiation therapy (IMRT) in patients with locally persistent nasopharyngeal carcinoma (NPC).

**Methods:**

Thirty-two patients with locally persistent NPC after full dose of IMRT were evaluated retrospectively. 3D-image-guided HDR-BT treatment plan was performed on a 3D treatment planning system (PLATO BPS 14.2). The median dose of 16 Gy was delivered to the 100% isodose line of the Gross Tumor Volume.

**Results:**

The whole procedure was well tolerated under local anesthesia. The actuarial 5-y local control rate for 3D-image-guided HDR-BT was 93.8%, patients with early-T stage at initial diagnosis had 100% local control rate. The 5-y actuarial progression-free survival and distant metastasis-free survival rate were 78.1%, 87.5%. One patient developed and died of lung metastases. The 5-y actuarial overall survival rate was 96.9%.

**Conclusions:**

Our results showed that 3D-image-guided HDR-BT would provide excellent local control as a salvage therapeutic modality to IMRT for patients with locally persistent disease at initial diagnosis of early-T stage NPC.

## Background

Nasopharyngeal carcinoma (NPC) is a radiosensitive disease, and radiotherapy is the standard therapy for non-disseminated NPC. A growing data showed that improvement in NPC patients treated with intensity-modulated radiotherapy (IMRT) was demonstrated in achieving excellent local tumor control, especially in the locally early-T stage patients with the least toxicity [1-4]. However, local persistence of disease remains a major hindrance to successful treatment, and the locally persistent NPC patient carries a risk of local recurrence if the salvaging treatment is not given, locally persistent disease was defined as relapsed tumor within 6 months of completion of primary radiotherapy, and local recurrence was failure beyond 6 months [5,6]. Kwong *et al*. [7] showed their experience in the patterns of failure in NPC patients after the completion of radiotherapy was associated with an increased risk of local failure. Among 803 NPC patients, fifty-five (6.8%) showed persistent disease and positive biopsies at 12 weeks post-radiotherapy with only 40% of 5-year local control rate. Likewise, Sze *et al*. and Yan *et al*. also showed that the local persistence of NPC post therapy was associated with an increased risk of local recurrence [8,9]. Therefore, aggressive and effective salvage treatment for locally persistent NPC is very important [10-13].

Various salvage treatment techniques have reportedly been effective for locally persistent NPC, including surgical nasopharyngectomy [14], brachytherapy [5], stereotactic radiosurgery [15,16], and three-dimensional conformal radiotherapy (3D-CRT) [17]. In our cancer center, routine 2D brachytherapy was given for salvaging NPC with biopsy-proven persistence before May 2005. Since then, 3D-image-guided high-dose-rate intracavitary brachytherapy (3D-image-guided HDR-BT) was used instead for this group of patients. 3D-image-guided HDR-BT is a new treatment method that uses PLATO BPS workstation (Nucletron B.V., Veenendal, the Netherlands). 3D-brachytherapy treatment planning is based on a set of CT, MR or UltraSound (US) images. Empty catheters are placed within the patient via meatus. Once the catheters are in place, a set of CT images is taken and the catheters are reconstructed on these images. Following the catheter reconstruction, the source positions and dwell times are planned and the plan is optimized [18-22]. This planning strategy enables us to build up the real time isodose distribution in all CT slices to increase accuracy of delivery of the prescribed dose. Previous studies have reported that external beam radiotherapy (ERT) combined with 3D-HDR-ICBT treatment was an effective treatment modality in cervical cancer [23,24], breast cancer [25], and prostate cancer patients [26], and so on. In 2010, Johanne *et al*. [27] compared the dose conformity of CT-based 3D high-dose-rate brachytherapy (3D-HDR-BT) and IMRT to deliver a boost to the prostate after external beam radiotherapy, they reported that CT-based 3D HDR-BT produced a more conformal plan for the boost to the prostate than IMRT.

Therefore, we conducted a retrospective study to evaluate the therapeutic benefit of 3D-image-guided HDR-BT modality for salvage treatment of locally persistent disease after intensity-modulated radiotherapy (IMRT) in patients with nasopharyngeal carcinoma (NPC).

## Methods

### Selection criteria for 3D-image-guided HDR-BT

This retrospective analysis recruited 32 NPC patients with locally persistence. The study was approved by the institutional reviewed board at Sun Yat-sen University Cancer Center. Locally persistent disease was defined as relapsed tumor within 6 months of completion of primary radiotherapy, and local recurrence was failure beyond 6 months [28]. Once proven pathologically by biopsy to have local persistence, each patient underwent physical examination, fiber-optic nasopharyngoscopy, chest X-ray, abdominal ultrasonography, and MRI/CT scan of the base of the skull, nasopharynx, and neck. Additional tests to evaluate the extent of disease included liver function tests, alkaline phosphatase, chest x-ray, liver or bone scans when indicated. 3D-image-guided HDR-BT was given when the following criteria were satisfied: 1). the persistent disease was biopsy-proven; 2). persistent tumors either confined to the nasopharynx or involving the upper choana with 5 mm nasal extension.

### Treatment methods and response assessment

**Figure 1:**
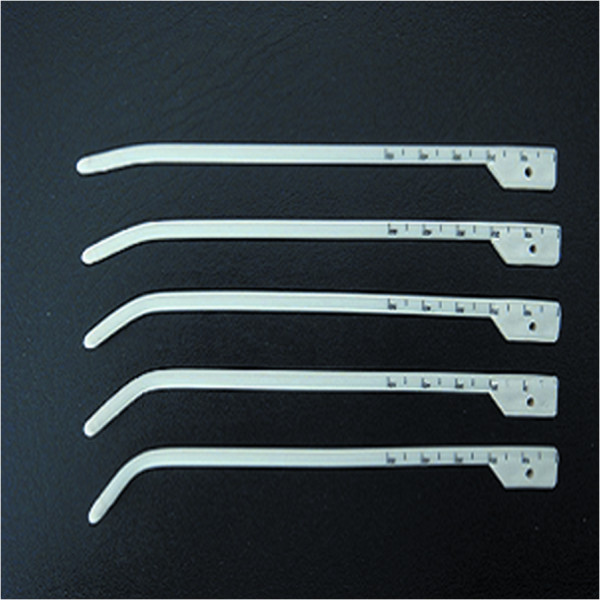
The customer-designed applicator for nasopharyngeal intracavitary brachytherapy.

**Figure 2:**
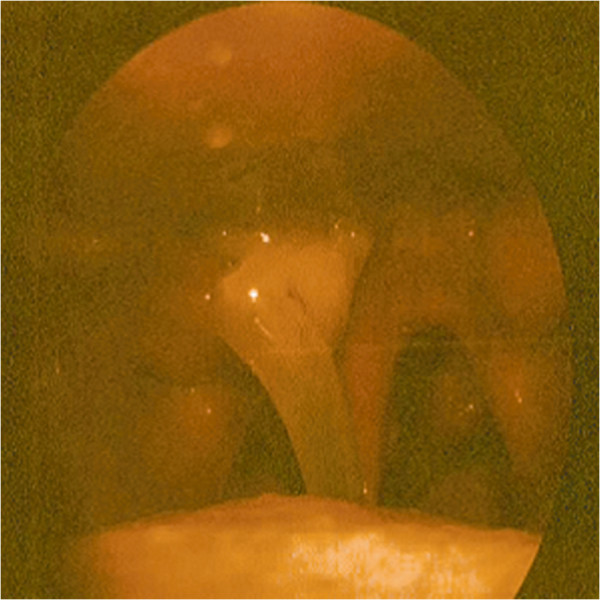
Customer-designed applicator for nasopharyngeal brachytherapy was placed at treatment positions near the persistent tumor under local anesthesia with fiberoptic endoscopic guidance via the inferior meatus.

**Figure 3:**
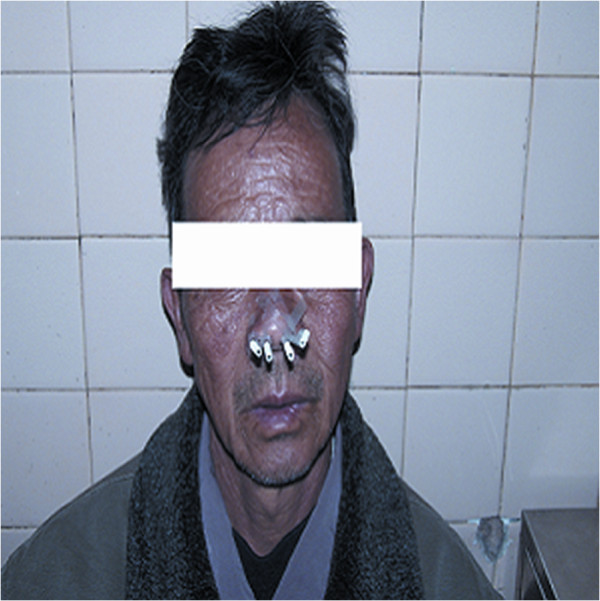
Four customer designed applicators for nasopharyngeal brachytherapy were immobilized.

**Figure 4:**
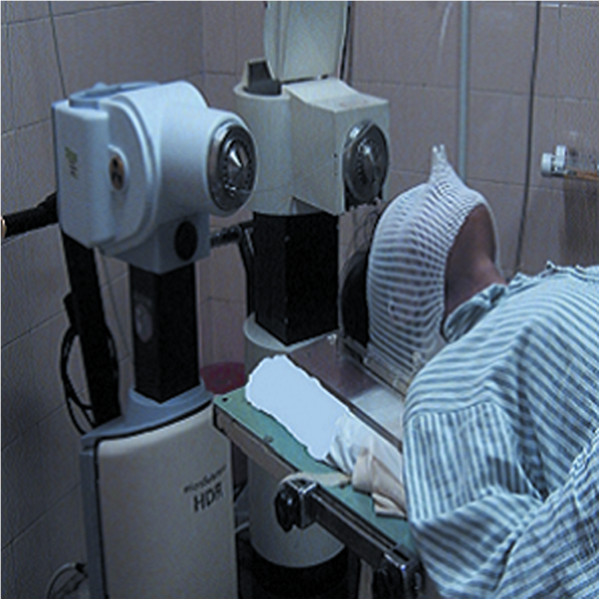
Patient was immobilized in the supine position with a thermoplastic mask.

**Figure 5:**
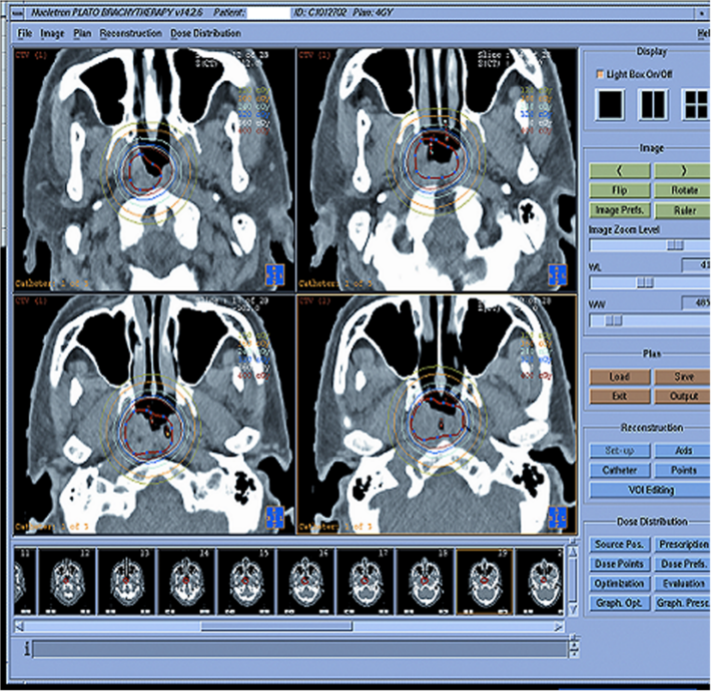
**Axial computed tomography (CT) image showed the applicators *in situ *for brachytherapy intracavitary with different isodose lines.**

The patients with locally persistence were treated with 3D-image-guided HDR-BT using a ^192^Ir source (microSelectron; Nucletron) when the selection criteria were satisfied at a median of 12 weeks after IMRT. The 3D-image-guided HDR intracavitary brachytherapy was delivered using a high-dose-rate (HDR) afterloading machine (microSelectron, Nucletron, Veenendaal, the Netherlands). All patients were treated under local anesthesia with fiberoptic endoscopic guidance via the inferior meatus to the treatment positions. Patients were immobilized in the supine position with a thermoplastic mask. All procedures were performed by the experienced radiation oncologist. Treatment plan was performed as follows: 1) two to four customer designed nasopharyngeal brachytherapy applicators (Figure 1) were placed at treatment position under local anesthesia with fiberoptic endoscopic guidance via the inferior meatus and the applicators were then immobilized (Figures 2, 3 and 4); 2) CT scanning using a 0.2 cm step to obtain 0.2 cm thick slices was performed and CT images were subsequently transferred to a 3D treatment planning system (PLATO PBS 14.2); 3) For all patients, gross tumor volume (GTV), clinical target volume(CTV) were assessed. To delineate these target volumes, MR images that were obtained were used as the reference. The GTV was determined by a radiation oncologist as the macroscopic extent of the persistent disease area. The CTV included the persistent disease area & a margin (0.5 cm) around the GTV; 4) following all target volumes defined and the applicators reconstruction, The PLATO PBS was used to calculate the dosimetry for a HDR ^192^Ir stepping source. The distance between each source step was 2.5 mm; 5) the dose optimization was done step by step manually. This planning strategy enables us to build up the real time isodose distribution in all CT slices to increase accurate delivery of the prescribed dose (Figure 5). Conformity index (CI=V_RI_/TV, where V_RI=_ reference isodose volume and TV= target volume) are calculated [29]. After approval of the plan, the treatment was delivered to the patients accordingly.

Treatment responses for all patients were evaluated according to the WHO response criteria [30]. Confirmation of response was assessed by nasopharyngoscopy, biopsy and MRI scans 12 weeks after the completion of the 3D-image-guided HDR-BT treatment. All acute and late radiotherapy toxicity was graded according to the criteria of the Radiation Therapy Oncology Group (RTOG) of the European Organization for Research and Treatment of Cancer (EORTC) [31].

### Follow-up and statistical analyses

All patients were seen and carefully evaluated by an experienced radiation oncologist. The duration of follow-up was calculated from the first day of brachytherapy to either the day of death or the day of last examination.

Survival rates were calculated based on the date of diagnosis of local persistence. The primary endpoint of this study (interval to the first defining event) were assessed: local failure free survival (LFFS), disease-free survival rate (DFS), distant metastasis-free survival rate (DMFS), and overall survival (OS). Actuarial survival was calculated according to the method of Kaplan-Meier [32].

## Results

### Patient populations and characteristics

Between May 2005 and December 2009, 32 consecutive patients with locally persistence received 3D-image-guided HDR-BT as salvage treatment 12 weeks after a full course of IMRT in our institute. Evaluation of treatment response and toxicity was made in single individual patient. The demographic details are shown in Table 1. The male/female ratio was 3.5:1 with 25 males and 7 females, and the median age was 44 y (range, 29~68 y). Histological examination showed that 96.9% of the patients had World Health Organization (WHO) Type II or III disease, 3.1% had WHO Type I disease.

**Table 1:** Characteristics of 32 NPC patients with locally persistent after IMRT

**Characteristic**		N	%
Age (years)	Median	44	
	Range	29 ~68	
Gender	Male	25	78.1
	Female	7	21.9
Histology	WHO type I	1	3.1
	WHO type II	6	18.8
	WHO type III	25	78.1
T stage	T1	6	18.8
	T2	12	37.5
	T3	12	37.5
	T4	2	6.2
N stage	N0	12	37.5
	N1	14	43.8
	N2	5	15.6
	N3	1	3.1
AJCC Stage	I	4	12.5
	II	12	37.5
	III	13	40.6
	IV	3	9.4
Concurrent chemo-radiotherapy	Received	6	18.8
	Not received	26	81.2

NPC: Nasopharyngeal Carcinoma; IMRT: Intensity-modulated Radiotherapy; WHO: World Health Organization; AJCC: the American Joint Committee on Cancer staging 7th edition (2011).

### Response to radiotherapy and survival

The 3D-image-guided HDR-BT was given as salvage treatment at median 12 weeks (range 10~16 weeks) after the primary treatment. Figure 5 shows the conformality of the dose distribution, the dosimetry planning was done based on CT images, the CTV was covered 100% of the prescription dose of 12 to 20 Gy (mean 16 Gy/4 fractions). All 32 patients achieved complete response (CR = 100%). Median time to complete regression was 1.0 months (range, 0.5~1.8 months).

**Figure 6:**
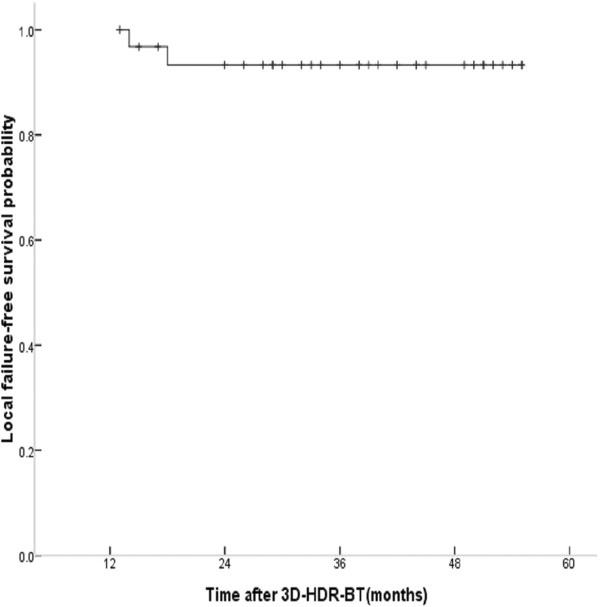
Actuarial local failure-free survival (LFFS) curves in patients with locally persistent NPC disease treated with the 3D-image-guided HDR-BT after IMRT.

With a median follow-up of 39 months, 7 patients developed local recurrence and/or distant metastasis (Table 2). Two patients developed locoregional failure, the one patient (T3N1, WHO III) had recurrence in parapharyngeal space at 14 months after completing treatment, and the other one (T4N2, WHO III) in the skull base at 16 months, respectively, they were successfully retreated. The 5-y actuarial LFFS rate was 93.8% shown in Figure 6. Further study showed that the patients with local persistence at initial diagnosis of early-T stage NPC had excellent 5-y LFFS rate (100%) comparing to those with advanced T stage NPC.

**Table 2:** Failure patterns in NPC patients with locally persistent disease

**Pt. No.**	**Sex /Age**	**AJCC T stage at initial diagnosis**	**AJCC T stage at relapse**	**Primary treatment**	**Second treatment for relapse**	**Time to relapse (mo)**	**Relapse site**
1	F/47	T3	rT1	IMRT 68 Gy	3D-HDR-ICBT	14	Parapharyngeal space
					16 Gy		
2	M/33	T4	rT2a	IMRT 68 Gy	3D-HDR-ICBT	16	Skull base
					12 Gy		
3	F/59	T2	rT1	IMRT 68 Gy	3D-HDR-ICBT	34	Bone
					12 Gy		
4	F/44	T1	rT1	IMRT 68 Gy	3D-HDR-ICBT	27	Bone
					12 Gy		
5	M/57	T1	rT1	IMRT 68 Gy	3D-HDR-ICBT	19	Neck nodal
					16 Gy		
6	F/62	T1	rT1	IMRT 68 Gy	3D-HDR-ICBT	24	Liver
					12 Gy		
7	F/42	T2	rT2a	IMRT 68 Gy	3D-HDR-ICBT	17	Lung(29 months died)
					20 Gy		

AJCC: American Joint Committee on Cancer staging system (2002); Pt. No.:patient number; F/M: female, male; IMRT: Intensity-modulated Radiotherapy; Chemo-IMRT: concurrent IMRT and chemotherapy; 3D-HDR-ICBT: Three-Dimensional CT-based high-Dose-Rate Intracavitary Brachytherapy. Time to relapse (mo): After second treatment(3D-HDR-ICBT) for relapse, the time, patients developed local recurrence and/or distant metastasis, is defined as Time to relapse (mo).

**Figure 7:**
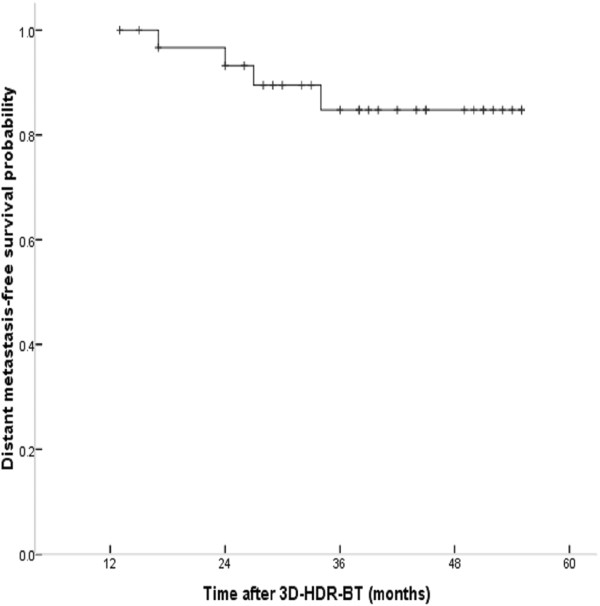
5-y actuarial distant metastasis-free survival (DMFS) curves in patients with locally persistent NPC disease treated with IMRT and 3D-image-guided HDR-BT.

Four patients developed distant metastases: bone metastasis in two patients at 27, 34 months respectively; liver metastasis in one patient at 24 months and lung metastases in one who died at 29 months after 3D-image-guided HDR-BT treatment. As shown in Figure 7. The actuarial 5-y DMFS rate after treatment was 87.5%.

**Figure 8:**
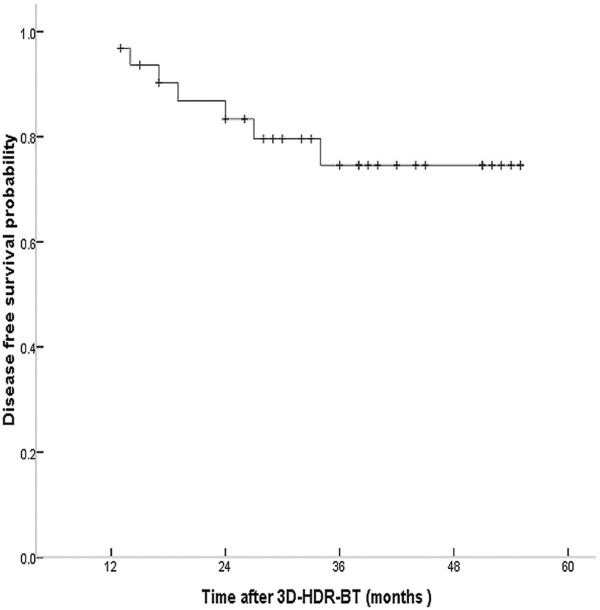
5-y actuarial disease-free survival (DFS) curves in patients with locally persistent NPC disease treated with the 3D-image-guided HDR-BT after IMRT.

One patient developed neck node recurrence at 19 months after 3D-image-guided HDR-BT treatment. The 5-y actuarial DFS rate was 78.1%, and the PFS curve is shown in Figure 8. In the present study, one patient died of lung metastasis at 29 months after 3D-image-guided HDR-BT treatment. The 5-y actuarial OS rate was 96.9%.

### Side effects and complications

The whole procedure of 3D-image-guided HDR-BT was generally well tolerated under local anesthesia. On follow-up examination, toxicity was mainly grade 1 or grade 2. The most common radiation-related complication was grade I or II xerostomia. No patient experienced beyond grade II xerostomia. Thirteen patients experienced grade I/II mucositis, which recovered to normal except a small white scar corresponding to the brachytherapy applicator position. Nasopharyngeal middle ulceration was observed in two patients with foul-smelling crust, one patient developed synechiae of the nasal mucosal linings at 23 months after treatment. The maximum oral aperture was measured in all 32 patients to assess temporomandibular joint dysfunction, and the mean pre- and post-treatment aperture was 4.5 cm and 4.0 cm, respectively. The difference between pre- and post-treatment aperture was not statistically significant. Total hearing loss, neck fibrosis limiting movement, and neurological complications (temporal lobe necrosis and cranial nerve palsy) were not observed in this study.

## Discussion

Recently, the use of 3D treatment planning system has increased in most radiotherapy facilities. This method allows for a better assessment of GTV and the definition and delineation of CTV compared with traditional approaches. A 100% isodose line was selected to cover the entire target as optimally as possible, then manual optimization on each CT slice was done interactively by dragging the 100% isodose line to cover the target volume as conformally as possible in this study. Our experience has shown that this CT-based-3D planning approach improved target volume delineation and optimal isodose coverage, and is more accurate and much easier than the conventional orthogonal film dosimetry.

In the current study, we treated 32 consecutive nonmetastatic NPC patients with local persistence disease using 3D-image-guided HDR-BT after IMRT. The whole procedure was well tolerated under local anesthesia. The results showed the 3D-image-guided HDR-BT was an effective treatment technique for local persistence, the actuarial 5-y LFFS rate was 93.8% with minimal toxicity. On subgroup analysis, the corresponding 5-y LFFS rates for T1, T2, T3, T4 at initial diagnosis were 100%, 100%, 91.7%, 50% respectively. The flexible customer designed applicators can be easily placed close to the nasopharyngeal mucosa resulting in a higher dose to the persistent lesion. With higher mucosal dose delivered by this technique, the benefit was gained in improving the local control of patient treated with this technique. This treatment results support our hypothesis that an adequate Salvage treatment to the locally persistence could compensate for primary treatment. In our study, 2 patients (T3 and T4 one each) with an initial complete response developed locoregional failure (one in parapharyngeal space and one in skull base) as assessed by nasopharyngoscopy, biopsy and MRI scans beyond 6 months at the completion of Salvage treatment. For advanced T stage NPC at initial diagnosis, the locally persistence may not be confined to the nasopharynx after IMRT. Due to its dosimetric limitations, 3D-HDR-BT therapy seemed to be not enough for patients with persistent disease beyond the area of nasopharynx.

Five patients developed relapse in other sites, one in the neck; the other 4 distant metastasis including one in the liver; 1 in the lung and 2 in the bone. 3D-image-guided CT-based HDR-BT did not improve the distant control rates as a salvage treatment. These results concurred with a previous study by Leibel SA *et al*. [33], which showed that nasopharynx tumors have a higher probability of micrometastatic dissemination at initial diagnosis, and concluded that the effect of local tumor control on survival cannot be determined without more effective methods to treat disseminated disease. Chang JT *et al*. [34] used ^18^F-fluorodeoxyglucose positron emission tomography (FDG-PET) and found that 11% of distant metastases in NPC patients were not discovered using the conventional staging workup (CWU). Based on this finding, they suggested that FDG-PET diagnosed stage M for NPC should be more accurate and sensitive than CWU. Due to its cost and availability, FDG-PET was not performed for each single patient in our study. Therefore, it was uncertain whether improved local tumor control by 3D-HDR-ICBT was associated with improved distant tumor control.

Due to its rapid dose fall-off, brachytherapy with Ir-192 to the locally persistent NPC post primary IMRT can deliver a high radiation dose to the target volume while sparing parotid glands and other vital structures, achieving higher local tumor control rate with relatively lower complications compared to external beam boost. The xerostomia syndrome was commonly seen as a long-term side effect in NPC patients when treated with primary radiotherapy. Our data showed patients with locally persistent NPC lesion experienced mild xerostomia when 3D-image-guided CT-based HDR-BT was used as salvage treatment to IMRT. Three patients experienced Grade-II mucositis could be ascribed to the high dose to the nasopharyngeal mucosa. During the long-term follow-up, the only complication related to brachytherapy was synechiae of the nasal mucosal linings in one patients, which can be treated easily by surgery, or adjust the loading pattern during the course of placing applicators to 3D-HDR BT.

At present, the optional gap time for salvage of locally persistent NPC patients after the completion of primary radiotherapy is still an issue. In this respective analysis, the mean gap period was 12 weeks. Zheng *et al*. [17] reported, the median of their gap period is also 12 weeks. Kwong *et al*. [7] recommended a confirmatory biopsy at 10 weeks before first salvage treatment. They analyzed the time course of histologic remission after radiotherapy in 803 NPC patients. Their results showed 6.9% of the total patients had residual disease at week 12, 16.3% had spontaneous remission observed in repeated biopsy after initial positive histologic proven, defined as delayed histologic remission; 76.8% had negative histologic features within 12 weeks after the completion of RT, defined as early histologic remission. They found that the delayed histologic remission is not a poor prognostic factor and the salvage treatment might be unnecessary. According to their study, some patients might have delayed histologic remission and the additional salvage treatment might be unnecessary in our series. In contrast, Withers *et al*. [35] revealed evidence of accelerated tumor regrowth in analysis nearly 500 oropharyngeal cancer patients with extensions of treatment from 5 to 8 weeks, a dose increment of about 0.6 Gy per day was required to compensate for tumor repopulation. Considering tumor repopulation between the completion of the first course radiotherapy and the salvage treatment, a shorter gap period could achieve higher local control. In our current series, the overall 5-y actuarial LFFS rate was 93.8%, and the excellent LFFS rate was also reported by Zheng *et al*. [17] 100%, 94.12%, 84.38%, 83.92% for stage T1,T2,T3,T4 disease using 3D-CRT salvage treatment at the short gap time period. Those results showed that early intervention for persistent NPC patients required a low salvaging radiation dose, obtained an excellent local tumor control with low rate of complications.

In conclusion, 3D-image-guided CT-based HDR-BT achieved excellent local tumor control rate as salvage treatment to primary IMRT for patients with locally persistent NPC disease, especially for those with T1-2 disease at the initial diagnosis. Xerostomia syndrome and other toxicities associated with this approach have decreased. Despite high locoregional control, distant metastasis remains main obstacle to the successful treatment for this group of patients; more effective therapy is warranted to improve the outcome.

### Consent (Adult)

Written informed consent was obtained from the patient for publication of this report and any accompanying images.

## Competing interests

The authors indicated no actual or potential conflicts of interest exist.

## Authors’ contributions

YFR - Primary author of manuscript and revisions. JX - Contributed to writing of manuscript and concept. WJY,YHG - Performed physics plans and assisted with manuscript. XPC - Concept of paper, contributed in writing manuscript and all revisions, BST, BXW- Concept of paper, contributed in writing manuscript and all revisions. All authors read and approved the final manuscript.
